# The First 1000 Days of PKU: A Narrative Review of Maternal PKU and Early Life Management After Positive Newborn Screening

**DOI:** 10.3390/nu18020199

**Published:** 2026-01-08

**Authors:** Elvira Verduci, Martina Tosi, Juri Zuvadelli, Sara Giorda, Giacomo Biasucci, Vincenzo Leuzzi, Marco Spada, Alberto Burlina, Carlo Dionisi Vici

**Affiliations:** 1 Department of Health Sciences, University of Milan, 20142 Milan, Italy; martina.tosi@unimi.it; 2 Department of Pediatrics, Vittore Buzzi Children’s Hospital, 20154 Milan, Italy; 3 Clinical Department of Pediatrics, San Paolo Hospital, ASST Santi Paolo e Carlo, 20142 Milan, Italy; juri.zuvadelli@asst-santipaolocarlo.it; 4 Department of Pediatrics, Metabolic Diseases, University of Turin, 10124 Turin, Italy; saragiorda.91@gmail.com (S.G.); marco.spada@unito.it (M.S.); 5 Pediatrics and Neonatology Unit, Guglielmo da Saliceto Hospital, 29121 Piacenza, Italy; g.biasucci@ausl.pc.it; 6 Department of Medicine and Surgery, University of Parma, 43121 Parma, Italy; 7 Unit of Child Neurology and Psychiatry, Department of Human Neuroscience, Sapienza University of Rome, 00185 Rome, Italy; vincenzo.leuzzi@uniroma1.it; 8 Division of Inherited Metabolic Diseases, Department of Women’s and Children’s Health, Reference Centre Expanded Newborn Screening, University Hospital, 35128 Padua, Italy; alberto.burlina@unipd.it; 9 Division of Metabolic Diseases and Hepatology, Bambino Gesù Children’s Hospital IRCCS, 00165 Rome, Italy; carlo.dionisivici@opbg.net

**Keywords:** phenylketonuria, phenylalanine, pregnancy, maternal PKU, newborn screening, breastfeeding, protein substitutes, complementary feeding, low-protein foods

## Abstract

**Background/Objectives**: The first 1000 days of life represent a critical window for growth and neurodevelopment, during which nutrition strongly influences brain development and metabolic programming. In phenylketonuria (PKU), dietary management is essential to prevent neurological impairment and later-life risk of non-communicable diseases (NCDs). This review examines current evidence on PKU from pregnancy through complementary feeding, highlighting the impact of nutritional strategies on neurodevelopmental and metabolic outcomes. **Methods**: This narrative review, following PRISMA guidelines, used a systematic search of PubMed and Scopus with defined PICO questions. Original research, reviews, and guidelines on PKU nutrition during the first 1000 days were included, emphasizing neurological and metabolic outcomes. **Results**: Articles addressed prenatal and postnatal factors in PKU. Optimised metabolic control in women with PKU is critical to prevent maternal PKU syndrome, reducing risks of miscarriage, congenital heart defects, microcephaly, and neurocognitive impairment. Pre-conception dietary management, frequent blood Phe monitoring, supplementation with Phe-free protein substitutes (PSs), micronutrients, and emerging pharmacological therapies support maternal and foetal health. Following newborn screening, early dietary treatment in infants with PKU maintains plasma Phe within safe ranges, promoting growth and neurodevelopment. Breastfeeding, combined with Phe-free infant PSs, is feasible, and complementary feeding should be introduced carefully. Frequent monitoring and tailored dietary adjustments, including second-stage PSs, support metabolic control, while data on gut microbiota remain limited. **Conclusions**: Early multidisciplinary interventions are crucial to optimise metabolic and neurodevelopmental outcomes during this window of opportunity. Further research is needed to address remaining gaps and optimise PKU management across the first 1000 days.

## 1. Introduction

The first 1000 days of life represent a critical window for rapid growth and development, during which individuals are particularly sensitive to environmental factors. According to the Developmental Origin of Health and Disease (DOHaD) theory, environmental exposures during critical periods of early development can induce long-term physiological and metabolic adaptations, potentially predisposing individuals to non-communicable diseases (NCDs) later in life [1,2]. As a significant and modifiable environmental factor, nutrition during early life plays a crucial role in foetal programming, infant growth and development, and the regulation of metabolic health later in life [3]. Maternal nutrition during early life plays a pivotal role in shaping foetal and infant development. During gestation, nutrient availability and maternal dietary status influence foetal growth and metabolic programming through the maternal–foetal interface [4]. Lactation represents a second critical phase within the first 1000 days, during which human milk provides nearly all nutritional requirements for the first six months of life and serves as a unique and irreplaceable source of nutrition, exerting long-lasting benefits on both maternal and infant health [5,6]. When breastfeeding is not feasible, infant formula serves as an alternative [7]; however, differences in protein content and composition compared to human milk have been associated with higher growth velocities among formula-fed infants [8]. After six months, the introduction of complementary foods (CFs) becomes essential to meet the increasing nutritional needs of the growing child [9].

In Phenylketonuria (PKU), OMIM: 261600, the main therapeutic approach is the dietary management based on restricted natural protein intake, Phenylalanine (Phe)-free Phe-free L-AAs and/or low-Phe protein substitutes (PSs), and special low-protein foods (SLPFs) [10,11]. During infancy, breast milk is recommended due to its low Phe content and beneficial bioactive compounds, while Phe-free PSs ensure adequate protein intake within metabolic tolerance. When breastfeeding is not possible, appropriate infant formulas are used to meet nutritional requirements until complementary feeding (CF) is introduced [10,11]. If untreated, PKU results in severe neurological impairment characterized by profound intellectual disability, seizures, motor dysfunction, and behavioural disturbances [12,13], while early-treated patients may still present structural and functional brain alterations [14] and may have an increased risk of NCDs later in life [15,16]. These observations highlight that optimised dietary management in the first 1000 days is crucial not only for immediate neurodevelopmental outcomes but also for long-term metabolic health. Older individuals with PKU show gut dysbiosis, underlining the potential importance of the microbiota in the first 1000 days and its possible role in mediating long-term NCDs risk [17].

This narrative review aims to critically examine the existing literature on PKU during the first 1000 days of life, encompassing pregnancy, lactation, and complementary feeding. To contextualize these findings, evidence from studies conducted in the healthy population will also be presented, providing a comparative perspective on the role of early nutrition. The focus is on understanding how nutritional management in the first 1000 days of life influences neurodevelopment and metabolic outcomes in infants, its potential implications for reducing the risk of NCDs later in life, and on providing an updated overview of current evidence and clinical practice.

## 2. Materials and Methods

This narrative review was conducted using a structured and transparent literature search informed by the PRISMA (Preferred Reporting Items for Systematic Reviews and Meta-Analysis) framework [18]. While PRISMA elements were adopted to improve clarity in reporting the search and selection process, the review does not represent a full systematic review or meta-analysis. The systematic literature search was performed exclusively for studies related to PKU, while the general population was not included in the systematic search. Instead, information on nutritional and developmental aspects of healthy populations was collected from authoritative sources such as international guidelines, position papers, review and original articles. No a priori protocol was registered (e.g., in PROSPERO), as this review was designed as a narrative synthesis rather than a systematic review. Nevertheless, the main steps of the search and selection process were predefined to ensure transparency and reproducibility. This methodological choice reflects the main objective of the review, which is to describe and synthesize evidence on nutritional management and outcomes in PKU during the first 1000 days, while referring to the general population only for comparison and context.

### 2.1. Data Sources and Search Strategies

A panel of Italian PKU experts was convened to identify the relevant nutritional aspects to be addressed in the research, focusing on pregnancy, lactation, and complementary feeding with the clinical significance. Subsequently, two PICO (Population, Intervention, Comparison, Outcome) strategies were developed to guide the systematic search and data extraction. The first topic, “From maternal PKU to a neonate with positive newborn screening,” addressed the narrative question: *“In pregnant women with PKU, do targeted nutritional interventions compared with standard care affect the neurological development and metabolic outcomes of the neonate?*” The second topic, “The First 1000 days: the postnatal phase, from birth to the second year of life,” addressed the narrative question: *“In neonates, infants and young toddlers with PKU during the first two years of life, do targeted nutritional interventions compared with standard care influence neurological and metabolic outcomes?”*. Full details of all PICO components for both topics are provided in Supplementary Table S1. The literature search was conducted using PubMed/Medline, and Scopus Databases, following the comprehensive strategy outlined in Supplementary Table S2. Studies were included if they were original research articles published in English, with no limitations on publication date. Both primary and secondary scientific evidence were considered, encompassing systematic reviews, consensus statements, guidelines, observational studies, case reports, and case series.

### 2.2. Identification of Relevant Studies

Study selection was carried out independently by four authors (blinded). Reviewers conducted the selection process independently, but were not blinded to study authorship or journal, consistent with the narrative nature of this review. Following the removal of duplicate records, all articles were screened based on their titles and abstracts. Studies were excluded if they were background or irrelevant articles, addressed an inappropriate dietary exposure or population, were of an unsuitable publication type, reported incorrect outcomes, or were published in a language other than English. Full-text screening was subsequently performed, and any disagreements regarding study eligibility were resolved through consultation with a third author.

### 2.3. Study Selection and Evaluation

Selected articles were fully analysed to extract information addressing the two PICO topics (Supplementary Table S1). The main outcomes of interest were identified as metabolic and clinical endpoints. For PKU, metabolic outcomes included stabilization of plasma Phe levels, correction of nutrient deficiencies, optimization of amino acid profiles, and mitigation of neurotoxic effects associated with elevated phenylalanine in PKU. Clinical outcomes encompassed growth and neurodevelopment, prevention of PKU-related neurological complications, improvement in overall health status, and support of optimal infant development. Detailed information is provided in Supplementary Table S1.

## 3. Results

**Figure 1:**
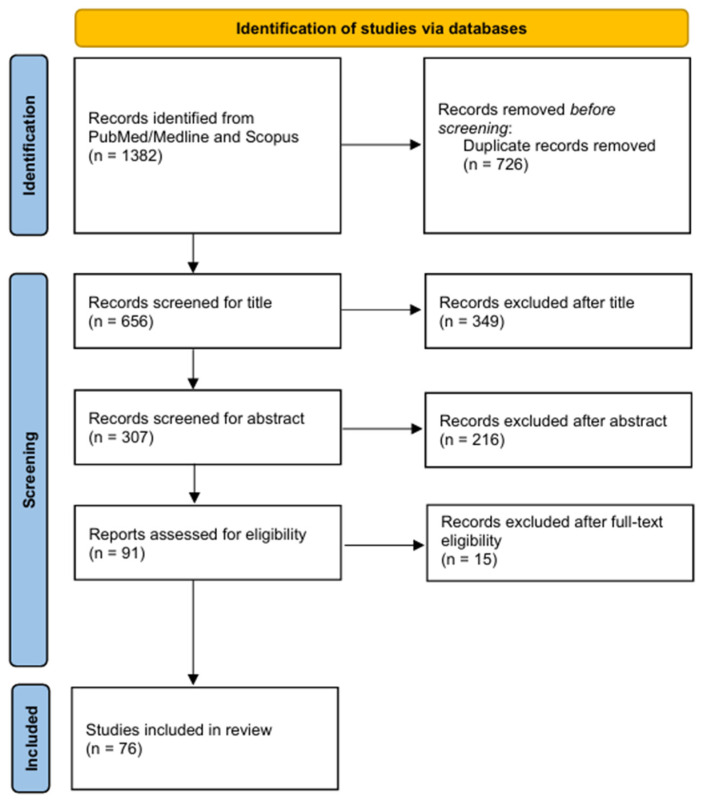
Flow diagram of the literature search process.

The initial systematic search identified a total of 1382 articles published between 1982 and 2025. Following the removal of duplicates, 656 records were screened based on titles and 307 based on abstracts, and 91 articles were subsequently assessed in full text. A total of 76 articles were ultimately included in the review. The review process is summarized in the flowchart shown in Figure 1. The results are organized into two main sections: prenatal factors, covering pregnancy up to birth, and postnatal factors, encompassing the period after birth. At the beginning of each section, a brief overview of key considerations for the general population was provided, addressing the key factors influencing infant health and development.

### 3.1. PKU and the First 1000 Days: From Maternal PKU to a Neonate with Positive Newborn Screening

#### 3.1.1. The First 1000 Days: Prenatal Phase (From Pre-Conception to Birth, 0–280 Days)—General Considerations for General Population

Parental environmental factors—such as diet, body composition, metabolism, and psychosocial influences—play a crucial role in shaping lifelong health and susceptibility to chronic diseases, as outlined by the DOHaD hypothesis [19]. The period around conception is particularly sensitive, as parental influences during gamete maturation and early embryonic development can have lasting effects, including increased risks of cardiovascular, metabolic, immune, and neurological disorders [20]. Maternal health and lifestyle during pregnancy, encompassing physiology, nutrition, and body composition (including excess fat mass and obesity), further affect long-term child well-being [21]. Adverse gestational conditions may predispose offspring to NCDs later in life, including hypertension, obesity, type 2 diabetes, atopic disorders, and certain cancers [22,23,24].

Optimal maternal nutrition during pregnancy is essential for foetal growth and development. Key nutrients include folate, choline, carotenoids, iodine, iron, vitamin D, and long-chain polyunsaturated fatty acids (LC-PUFAs), which prevent congenital defects and support maternal and foetal health [25]. Knowledge gaps remain regarding the effectiveness of periconceptional interventions, including multivitamin and micronutrient supplementation such as vitamin B12 [26], underscoring the need to tailor nutrition to individual maternal dietary patterns.

Exposure to parental tobacco smoke during pregnancy and lactation is consistently associated with adverse offspring outcomes, including preterm birth, foetal growth restriction, low birth weight, sudden infant death syndrome, neurodevelopmental and behavioural problems, obesity, hypertension, type 2 diabetes, impaired lung function, asthma, and wheezing [27]. Smoking also increases obstetric complications and in utero health risks [28]. Addressing barriers to cessation is essential, and further research on medications and e-cigarette use during pregnancy is needed.

Regular, supervised physical activity during pregnancy benefits both mother and child [29]. It improves cardiovascular health, controls weight gain, reduces gestational diabetes and hypertension risk, and supports physical and psychological well-being. For the foetus, maternal exercise promotes healthy growth, neurodevelopment, lower fat mass, and is associated with shorter labour and fewer operative deliveries. Despite these benefits, many women reduce activity, increasing metabolic and obstetric risks. Promoting prenatal exercise is therefore an important preventive and therapeutic strategy [30].

Obesity is common in women of reproductive age and poses risks for maternal and child health. It disrupts hormonal balance, increases insulin resistance and chronic inflammation, and may induce epigenetic changes affecting ovarian function and endometrial receptivity [31]. Even modest increases in maternal BMI raise the risk of foetal death, stillbirth, and neonatal, perinatal, and infant mortality [32]. Maternal obesity is linked to infertility, miscarriage, congenital anomalies, gestational glucose intolerance, foetal overgrowth, and higher caesarean rates [33]. Postpartum, obese women face increased thromboembolism, depression, and breastfeeding difficulties, while their children have higher birth fat and risk of childhood obesity. Excess gestational weight gain amplifies these risks and contributes to long-term cardiometabolic complications [34].

Gestational diabetes mellitus (GDM), often alongside maternal obesity, increases pregnancy risks [35]. Both conditions involve insulin resistance, disrupted carbohydrate and lipid metabolism, and chronic inflammation, contributing to placental dysfunction and oxidative stress [36]. Women with obesity and GDM face higher rates of hypertensive disorders, preterm delivery, caesarean section, and large-for-gestational-age or macrosomic infants [37]. Effective management reduces adverse outcomes. Type 1 diabetes further raises congenital malformation and perinatal mortality risks [38]. Optimal glycaemic control is crucial, aided by continuous glucose monitoring, insulin pumps, closed-loop systems, ultra-rapid insulin analogues, and comprehensive patient education [39].

#### 3.1.2. Nutritional Approach in Maternal PKU

Optimised metabolic control during pregnancy in women with PKU is essential to prevent the maternal PKU (MPKU) syndrome, whose fetotoxic effects of hyperphenylalaninemia include intrauterine growth retardation, low birth weight, microcephaly, developmental delay, intellectual disabilities, congenital heart disease and cleft lip and palate [40]. Historical experience on pregnancy in PKU revealed that over 90% of mothers with uncontrolled hyperphenylalaninemia had intellectually disabled offspring [41]. Cumulative data from a large cohort of pregnant PKU mothers show that early pre-conception dietary intervention improves metabolic control. This is associated with a lower incidence of miscarriages. On the other hand, initiation of dietary treatment during pregnancy with poor metabolic control is associated with high rate of congenital heart disease, intellectual disability, and microcephaly [42]. Suboptimal metabolic control during pregnancy has been estimated to be associated with miscarriages in about 7% of offspring, also associated with low-rated cognitive tests at 4 and 6 years of age [42,43]. These findings highlight the importance of educating women with PKU to follow a lifelong diet, particularly to optimise metabolic control before conception. Pregnancy should be carefully planned, including optimising dietary management, biochemical and clinical follow-up, and metabolic control in the months prior to conception [44].

To date, blood Phe concentration 120–360 µmol/L is considered safe during pregnancy [11]. Structured education and support for women with PKU and their partners are therefore essential to maintain safe Phe levels and prevent the maternal PKU syndrome, ensuring better outcomes for offspring [45]. Women with PKU require careful monitoring of blood Phe, at least weekly pre-conception and twice weekly during pregnancy, alongside effective contraception to avoid unplanned pregnancies [11]. Optimised metabolic control is essential to prevent the MPKU syndrome and ensure favourable outcomes for offspring [45]. Phe tolerance increases during pregnancy, particularly from the second trimester, due to foetal anabolism and Phe hydroxylation. In the case of a non-PKU foetus, Phe tolerance can triple in the third trimester, whereas this is not observed with a foetus affected by PAH deficiency [46].

Nutritional management is based on natural protein restriction according to individual Phe tolerance, supplementation with Phe-free L-AAs enriched in micronutrients, and SLPFs [10]. Despite dietary management, energy and nutritional requirements remain similar to healthy pregnancies, and insufficient total protein intake is associated with suboptimal foetal growth and increased risk of congenital heart disease [47]. Protein requirements increase during pregnancy, with an additional 20% of Phe-free/low-Phe PSs recommended in trimesters 1–2 and a further 30 g/day in trimester 3, primarily from natural protein; lactation requires an extra 20 g/day [10,11]. Tyrosine intake should be ≥6 g/day, provided through PSs, and nausea or vomiting should be managed to ensure adequate energy and protein intake [10,11]. European guidelines in 2017 used to recommend a total protein intake of ≥70 g/day, combining natural protein and PSs [48]. PSs include traditional Phe-free L-AAs, sometimes enriched with large neutral amino acids (LNAA) or delivered as prolonged-release PSs, and glycomacropeptide (GMP) formulations, which allow optimal metabolic control without significant Phe intake [49,50]. Specific protein substitutes for use during pregnancy and lactation are currently limited on the market.

Micronutrients are crucial for maternal and foetal health. According to 2025 PKU Guidelines, all women should receive at least 400 μg/day of folic acid when planning pregnancy and throughout the first 12 weeks of gestation, irrespective of the folic acid content of supplemented Phe-free or low-Phe PS [11]. In addition, a daily intake of ≥200 mg of Docosahexaenoic acid (DHA) should be provided to all women with PKU who are planning pregnancy and during pregnancy. DHA [10,11]. Optimal neurodevelopment is a key concern in PKU, as the dietary regimen excludes natural sources of DHA. In healthy pregnancies, folic acid is recommended during the first and second trimesters [51,52], while DHA becomes particularly important in the second and third trimesters [53,54]. In women with PKU, who follow a non-omnivorous diet, combined supplementation with folic acid and DHA is advised until the end of the second trimester, with DHA continued thereafter. Given the potential for higher DHA requirements and the possible interaction between folic acid and DHA metabolism [55,56], PSs for pregnancy should provide folic acid levels appropriate for pregnancy to prevent excessive intake and support optimal DHA utilisation. Key nutrients, including iron, iodine, zinc, selenium, vitamin D, and LC-PUFAs, should be assessed pre-conception and early pregnancy, with further monitoring in later trimesters if dietary adherence is sub-optimal or deficiencies are identified [10,11,57,58,59,60,61]. Vitamin B12, important for neurodevelopment, may be deficient in pregnancy, but in women with PKU adhering to the diet it is provided by PSs. Overall, these early nutritional strategies are associated with favourable long-term clinical outcomes in offspring, including reduced risk of maternal PKU syndrome, congenital malformations, and neurocognitive impairments.

Women with PKU often face challenges in maintaining good nutritional status, particularly during their reproductive years. While BMI is generally similar to that of healthy women, those with classical PKU or poor metabolic control tend to be overweight or obese, which can affect pregnancy outcomes [62,63]. Disordered eating behaviours, food neophobia, and dislike or gastrointestinal intolerance to PSs can compromise nutrient intake, while dietary patterns high in simple sugars and low in fibre may negatively influence the gut microbiota and long-term metabolism [62,63,64,65]. Currently, no studies have specifically investigated the gut microbiota of pregnant women with PKU, but such research could clarify its role in maternal metabolism and foetal development.

Infants without PKU born to women with PKU can metabolise the Phe in maternal breast milk without difficulty [11]. Even if the infant has PKU, breastfeeding is possible when combined with a Phe-free infant formula. In women with PKU, the Phe content of breast milk is highest immediately after birth, but it typically decreases to 90–130 mg/100 mL in the following days [48]. However, overall data on breast milk Phe content in relation to maternal blood Phe control remain limited.

Immediate metabolic and growth outcomes are well described, but evidence on the long-term effects of these early nutritional strategies is limited. Observational studies suggest that good metabolic control during pregnancy and early infancy may reduce the risk of neurodevelopmental impairments and support favourable growth. However, further longitudinal research is needed to clarify the impact on long-term metabolic health and the potential risk of NCDs.

#### 3.1.3. Pharmacological Approach in Maternal PKU

Women with PKU are encouraged to breastfeed their offspring, as benefits of breastfeeding are expected to be similar to those in general maternal and neonatal populations. Data correlating maternal metabolic control and breast milk composition are scanty, although breast milk composition is known to be only marginally influenced by environmental conditions [66,67].

Sapropterin dihydrochloride (BH4) treatment has been suggested proposed as a complementary treatment to diet in BH4-responsive pregnant women with PKU. During pregnancy, Sapropterin was well-tolerated and associated with good metabolic control and uncomplicated offspring outcomes [68,69]. In BH4-responsive patients with PKU, pharmacological treatment often enables a substantial increase in natural-protein intake and relaxes dietary restrictions [70]. This liberalisation may compromise diet quality if not carefully monitored, as it has been associated with reduced intake of essential micronutrients and LC-PUFAs, potentially increasing the risk of nutritional deficiencies, as well as a lower intake of pre- and probiotics that positively impact the gut microbiota [71,72]. Thus, while pharmacotherapy allows greater natural protein intake, careful monitoring of overall dietary quality remains essential to avoid micronutrient gaps. As there is no clear evidence that this improves anthropometric outcomes, nutritional biomarkers, or quality of life [73], greater attention to dietary composition and micronutrient intake is required during pregnancy.

Pegvaliase, a recent enzyme replacement therapy for PKU, has been shown to effectively improve metabolic control in adult patients allowing an unrestricted diet [74]. As this treatment is currently not approved during pregnancy, current experience is scanty and only based on anecdotal data. Revision of data from women on Pegvaliase who chose to continue this treatment throughout their 14 pregnancies revealed uncomplicated outcomes. Six females and eight males were born without congenital anomalies and all offspring had normal growth parameters. In this small cohort, four of eleven infants (excluding triplet pregnancies) were delivered preterm (36%, with respect to 12% in the general population), and a single 8 weeks-miscarriage was reported [75].

Evidence on pharmacological therapies such as BH4 and Pegvaliase during pregnancy remains limited, and further studies are needed to confirm their safety and efficacy.

The use of large neutral amino acids (LNAAs) is not recommended during pregnancy due to limited evidence on their effects on foetal growth and neurological development. Furthermore, LNAAs do not reduce Phe levels sufficiently to prevent potential teratogenic effects, and safety data in pregnant women are lacking [76].

### 3.2. The First 1000 Days: Postnatal Phase, from Birth to Second Year of Life

#### 3.2.1. Infant Feeding (0–6 Months): Breastfeeding and Formula Feeding—General Considerations for General Population: State of the Art

Breastfeeding is essential for infant survival, nutrition, development, and maternal health. The World Health Organization (WHO) recommends exclusive breastfeeding for six months, followed by continued breastfeeding with complementary foods for up to two years or longer [77,78]. Human milk provides nutrients and bioactive components, antibodies, human milk oligosaccharides (HMOs), extracellular vesicles, microRNAs, and commensal or probiotic bacteria, that support immunity, gut colonization, growth, and neurodevelopment [79]. Milk composition is dynamic and influenced by maternal, infant, and physiological factors, with evidence suggesting maternal body composition plays a key role [80]. Early, uninterrupted skin-to-skin contact and non-nutritive suckling methods for expressed milk are recommended, especially for preterm infants [78].

While breastfeeding remains the gold standard, formula feeding is a common alternative when breastfeeding is not possible or contraindicated [81]. Infant formulas (IFs) re specially formulated to meet infants’ nutritional needs until complementary foods are introduced, in accordance with Commission Delegated Regulation (EU) 2016/127 [82]. Evaluation of IFs should consider not only macronutrient quantity and quality, proteins, carbohydrates, lipids, but also immunomodulatory components, gut microbiota modulators, LC-PUFAs, and nucleotides [83].

A European randomized trial shows that higher protein content in infant formulas promotes greater weight gain in the first two years without affecting linear growth, while stimulating the IGF-I axis and insulin secretion, particularly in the first six months [84]. Female infants exhibit stronger endocrine responses than males, though without enhanced growth. Elevated early protein intake may accelerate weight gain and influence metabolic pathways in a sex-modulated manner, increasing later overweight risk [85]. Metabolomic analyses reveal elevated BCAAs and short-chain acylcarnitines in high-protein-fed infants, suggesting saturated BCAA degradation, inhibited β-oxidation, early fat deposition, and accelerated weight gain [86]. Long-term data indicate that higher protein intake in infancy affects BMI trajectories and raises the risk of childhood overweight, highlighting the need to carefully regulate protein in IFs [87].

Human milk lipids provide 45–55% of total energy in early infancy and are essential for growth, development, and metabolic regulation. Predominantly triglycerides, they supply PUFAs and LC-PUFAs vital for membrane formation, cell signalling, and neural development [88]. Although IF lipid composition has improved, it still differs from human milk in composition and structure. Per EU Regulation 2016/127 [82], DHA must range 20–50 mg/100 kcal, total LC-PUFAs ≤ 2% of fat, Arachidonic acid (ARA) ≤ 1% if added, Eicosapentaenoic acid (EPA) ≤ DHA. ARA is not mandated by the EU Regulation. Pre-regulation studies show LC-PUFA–supplemented formulas achieve plasma DHA levels comparable to breastfed infants, while non-supplemented formulas yield lower DHA, reflecting limited endogenous conversion [89]. Given the crucial role of LC-PUFAs in early development, recent expert opinion [90] recommends including ARA in infant formulas at levels comparable to or exceeding those of DHA (around 0.3–0.64% of total fatty acids). PUFAs supplementation also raises plasma ALA and serum folate, benefiting micronutrient balance [89]. Key differences from breast milk include LC-PUFA content, fatty acid positional distribution, cholesterol, and complex lipids, which may influence growth, metabolic programming, and long-term health.

Infant gut microbiota is shaped by gestational age, delivery mode, early care environment, and feeding type [91,92]. Breastfeeding promotes *Bifidobacterium* dominance, supporting saccharolytic bacteria and short-chain fatty acids (SCFAs) production from HMOs [92]. Formula-fed infants show greater microbial diversity, with higher abundances of opportunistic taxa such as Enterobacteriaceae, Bacteroidaceae, and Clostridiaceae [93]. Delayed or inadequate microbiota maturation is associated with impaired growth and early developmental outcomes. ESPGHAN has highlighted the potential role of bioactive components in shaping the gut microbiota of formula-fed infants, suggesting that future formulations may need to include such ingredients to better support gut microbiota development [94].

#### 3.2.2. Complementary Feeding and Early Diet (6–24 Months)—General Considerations for General Population: State of the Art

The period from weaning to two years is crucial for physical, cognitive, social, and behavioural development [95]. Complementary feeding (CF) transitions infants from exclusive milk (breast or formula) to a varied diet of liquids and solids [96], complementing rather than replacing milk [97]. WHO recommends exclusive breastfeeding for six months, with CF beginning thereafter while breastfeeding continues [98]. EFSA and ESPGHAN suggest initiating CF between 17 and 26 weeks, tailored to infant readiness [99,100].

Complementary foods (CFs) include purees, mashed or lumpy textures, finger foods, and other suitable beverages [98,99,100]. They should provide adequate energy, protein, and essential micronutrients, particularly iron, iodine, zinc, calcium, vitamins A and C, and folate, to support growth and brain development [101,102], while low-nutrient beverages such as tea, coffee, and sugary drinks should be avoided [103]. CFs must be hygienically prepared, free from pathogens, toxins, chemicals, bones, or hard pieces [101,102].

LC-PUFAs are critical for brain and visual development [104]. Breastfeeding provides LC-PUFAs naturally, and CFs help maintain adequate levels as dietary needs increase [105,106,107]. Early feeding habits and flavour exposures influence long-term dietary preferences, and inappropriate CF timing or inadequate nutrition can increase obesity, diabetes, cardiovascular disease, and developmental delay risks [108].

CFs also support psychosocial development by integrating infants into family meals, promoting oral-motor skills, and fostering healthy eating behaviours [109]. Timing varies by context: in high-income countries, earlier weaning (4–6 months) may be indicated for high-growth or iron-deficient infants [99,110], whereas WHO recommends initiating CF at six months, especially in low- and middle-income settings to reduce infection risk [111]. Very early CF (≤4 months) may increase overweight risk [112], and excessive protein intake during CF can affect growth patterns [96].

Exposure to varied textures is essential; delaying lumpy or solid foods beyond nine months can lead to feeding difficulties and reduced acceptance of healthy foods [113,114,115]. Early introduction of allergenic foods, from around four months in high-risk infants, may promote immune tolerance [116,117,118,119].

Responsive complementary feeding, where caregivers attend to infants’ hunger and satiety cues, provides a positive eating environment and supports appetite regulation and healthy dietary habits [100]. Parenting style also shapes the child’s relationship with food [120,121].

The introduction of CFs is a critical phase for gut microbiota development. Transitioning from a milk-based diet to CFs within the first year shapes microbial composition [122]. Timing of CF and breastfeeding duration influence microbiota diversity and SCFAs levels, which affect metabolic regulation and body weight. CF introduction promotes fibre-degrading bacterial families, including Lachnospiraceae, Ruminococcaceae, and Bacteroidaceae [123,124], increasing SCFAs production. Both early and late CF introduction may disrupt normal gut microbiota maturation [125,126].

In conclusion, complementary feeding from 6 to 24 months is a holistic process influencing developmental, behavioural, and emotional outcomes. It shapes food preferences, social habits, and long-term health. While global guidelines offer frameworks, CF should be tailored to each child’s developmental readiness, family environment, and socio-economic context.

#### 3.2.3. A Neonate with Positive Newborn Screening for PKU

Phenylketonuria is identified through newborn screening (NBS), which should be implemented in all European countries [11]. NBS requires a well-organized infrastructure, with blood samples collected within the first days of life and analysed in specialized laboratories. Low-income or smaller countries may rely on NBS facilities in other nations. Infants with a positive NBS result should be promptly referred to a specialized metabolic center for diagnostic confirmation, treatment initiation, and long-term follow-up. Treatment should begin when the plasma Phe concentration detected through NBS exceeds 360 μmol/L and must be initiated as early as possible, ideally within the first 10 days of life [11]. Early treatment is critical: evidence demonstrates that Phe levels > 360 μmol/L during the first month of life are associated with reduced childhood IQ, and each four-week delay in starting therapy may lead to an approximate decrease of 4 IQ points, although a precise “window of vulnerability” within the first month has not yet been clearly defined [127,128].

Patient education is a cornerstone of effective PKU management. Encouraging self-management skills that support stable metabolic control, and positive social development is essential. Practically, caregivers should help children acquire age-appropriate knowledge about PKU and its treatment from the earliest stages of life, and should also involve siblings and other family members in this learning process [129]. Structured educational interventions directed at caregivers are a critical component of early PKU management. Clinical evidence indicates that targeted training programs, particularly when combined with nutritional support, can improve phenylalanine control in affected children. These findings suggest that caregiver competence exerts a direct influence on achieving and maintaining optimal metabolic outcomes [130]. Furthermore, observational studies suggest that a greater understanding of the dietary principles of PKU, along with perceiving the diet as health-promoting, are associated with improved adherence to nutritional treatment. These findings underscore the pivotal role of education in supporting the day-to-day management of the condition [131]. Finally, education directed at parents, particularly during early childhood, is associated with improved dietary management. Indeed, it has been reported that the level of maternal knowledge significantly influences the child’s metabolic control [132].

In addition to providing nutritional management and educational support, metabolic centres play a dual role by confirming diagnosis through molecular genetic testing and by managing therapy, which may include dietary treatment and/or pharmacological options such as Sapropterin dihydrochloride (BH4). Although dietary treatment remains the first-line therapy, therapeutic options have expanded, and cofactor therapy may also be used in newborns who demonstrate responsiveness. It is estimated that 25% to 50% of infants with PKU may benefit from this approach [133]. European guidelines underscore that genotype is a key determinant of metabolic phenotype and responsiveness to tetrahydrobiopterin (BH4) [11]. Therefore, all infants should be evaluated for potential BH4 responsiveness, either through molecular analysis or via a BH4 loading test. Individuals responsive to BH4 typically carry PAH variants associated with mild to moderate phenotypes, whereas defects in genes involved in BH4 metabolism account for only 1–2% of all cases of hyperphenylalaninemia [134]. Data from the BIOPKU database show that allelic phenotype values (APV) predict BH4 responsiveness in approximately 71% of tested patients, supporting the routine use of genotyping in individuals diagnosed with hyperphenylalaninemia [135]. Supplementation with exogenous BH4 can partially or fully restore PAH activity, depending on the allelic variant and the degree of structural destabilization [136].

#### 3.2.4. Nutritional Considerations in PKU Infants and Toddlers (First Two Years of Life)

The dietary management of PKU in early life aims to reduce Phe intake by adjusting the amount of breast milk or standard IFs according to the infant’s individual metabolic tolerance, while maintaining metabolic control through weekly measurements of blood Phe concentrations [10]. Breast milk is widely recognized as the primary source of nutrition for healthy infants during the first two years of life. Its protein content is relatively low (on average 0.9–1.2 g/dL in mature term milk) and is characterized by a low protein-to-energy ratio, which makes it particularly suitable for infants with PKU [137,138]. Although randomized trials are lacking, observational evidence indicates that continued breastfeeding, supplemented with a Phe-free infant PS, is safe and may provide metabolic, growth, and neurodevelopmental benefits in infants with PKU [139]. At present, there is no universally accepted criterion for performing a washout period, defined as a temporary diet with zero phenylalanine intake. Empirically, a Phe-free diet, when adequately supplemented with energy and amino acids, results in a decrease in plasma Phe of approximately 400 μmol/L per 24 h. A washout period may be useful to shorten the duration of elevated plasma Phe levels. However, it should not be extended beyond 24–72 h to prevent hypophenylalaninemia. Generally, this approach is reserved for situations in which plasma Phe concentrations reach particularly high levels (e.g., >1000 μmol/L). A recent study further demonstrated that breastfeeding represents the optimal source of intact protein for infants under six months of age with PKU or hyperphenylalaninemia, being associated with better metabolic control (lower plasma Phe levels) and adequate nutritional status compared to formula feeding or mixed feeding [140]. Several methods have been described for administering defined amounts of human milk according to the infant’s individual phenylalanine tolerance. The main approaches include:

1. Administering a pre-measured volume of expressed breast milk prior to a Phe-free PS;
2. Administering a pre-measured volume of Phe-free PS followed by breast milk offered ad libitum until satiety;
3. Administering a pre-measured volume of Phe-free PS followed by time-controlled breastfeeding;
4. Providing alternate feeds of HM and Phe-free PS throughout the day, with progressive titration of milk volumes and adjustment of feeding frequency.

A study evaluating breastfeeding duration demonstrated that the method consisting of administering a pre-measured volume of Phe-free infant PS followed by ad libitum breastfeeding is effective in maintaining good metabolic control, while allowing breastfeeding to continue for a duration comparable to that observed in the general population [141], but this approach was not confirmed in a recently published systematic review [142]. Overall, evidence on combining breastfeeding with Phe-free PSs is limited and heterogeneous, and no definitive conclusion can be drawn on the best method. Choices should consider metabolic tolerance, feasibility, and the goal of maintaining breastfeeding, highlighting the need for individualised planning and close monitoring.

Beyond the quality of intact protein sources such as breast milk, attention should also be given to the composition and quality of synthetic PSs. A recent study [143] has highlighted that infant PS differ markedly in their formulation in terms of energy and protein content, and consequently in their protein-equivalent (P.Eq.) to energy ratio, but also in the inclusion of functional components such as EPA, FOS, GOS, synthetic HMOs, lactose, and nucleotides. Main issues included variability in energy and P.Eq. content; all products contained DHA and ARA, while EPA, FOS, GOS, synthetic HMOs, lactose and nucleotides were inconsistently present [143]. Lactose supports neurodevelopment via galactose and promotes a healthy gut microbiota [144,145]. Essential fatty acids further support growth, cognitive, and neurological development [146], while bioactive components enhance gut microbiota balance and immune function [147] potentially reducing the risk of NCDs.

Furthermore, only a single type of infant formula for PKU is available, with no stage-based follow-on formulas (e.g., stage 2, 3) comparable to those provided for healthy infants, particularly with regard to the P.Eq. to energy ratio and the content of essential micronutrients.

In most European centres for PKU, the initiation of complementary occurs slightly earlier than in the general population, typically between 17 and 26 weeks of age. The infant’s ability to accept foods other than milk does not differ from that of healthy peers; the progression toward more solid textures, the gradual reduction in formula intake, and the acquisition of self-feeding skills follow a comparable timeline [148]. The first complementary foods introduced are generally fruits and vegetables with a low Phe content (≤75 mg/100 g). Initially offered once daily, these foods are preferably given after a feeding of Phe-free infant PS or breast milk/standard infant formula, to avoid reduced appetite for the milk portion. Given that low-Phe foods are often also low in energy density, caloric intake should be carefully monitored and adjusted if necessary to ensure appropriate growth [10]. To ensure an adequate protein intake, it is necessary to introduce a more concentrated PS that provides a high density of protein equivalents in a small volume. The PS is intended to be combined with infant formula, covering approximately 50% of the total protein requirement at 7–8 months of age, and to gradually replace it completely by around two years of age [149].

The age at which the second-stage PS is introduced varies across Europe, and in some Southern European countries this introduction tends to be delayed [150]. This may be partly related to the fact that in some Southern European centres a lower total protein intake is recommended [151]. Conversely, early introduction of the second-stage protein substitute in spoonable or semi-solid form appears to promote better acceptance [152]. It is important to assess the micronutrient content of second-stage protein substitutes, as variations between products may affect the nutritional adequacy of the diet for children with PKU. A recent global survey of 106 healthcare professionals from 32 countries examined practices for transitioning protein substitutes in children with PKU [153]. Infant formula is typically discontinued between 1 and 2 years, but timing and types of substitutes vary widely. Product quality, tolerance, and practical barriers such as taste, texture, and access are major considerations [153].

Regarding complementary SLPFs, although specialised weaning products are available, there are relatively few age-appropriate products for children with PKU under two years of age.

During the first two years of life, children with PKU require strict dietary control to maintain safe plasma Phe concentrations while supporting normal growth and neurocognitive development. Because natural protein intake is severely restricted, Phe-free PSs also provide essential nutrients; however, micronutrient status may appear suboptimal in some patients, particularly with respect to DHA, iron, zinc, selenium, vitamin B12, folate, and vitamin D [154,155,156]. Despite recommendations for routine biochemical follow-up, specific guidance on micronutrient supplementation during early infancy remains undefined.

Complementary feeding is generally well accepted, although this represents a particularly sensitive period for mothers, who may experience anxiety and reduced gratification compared with mothers of unaffected children. Overall, maternal anxiety regarding the child’s refusal of PS has been observed to increase over time, peaking between 12 and 24 months of age. In PKU, the period between 12 and 18 months is identified as a critical stage during which mothers report higher levels of anxiety and stress during feeding, coinciding with elevated blood phenylalanine concentrations likely associated with teething, intercurrent illnesses, and the child’s developing independence. These aspects should be carefully considered in order to provide targeted support to families [157]. Dietary treatment, whether used alone or in combination with pharmacological therapy, must ensure that blood Phe concentrations are maintained within the recommended target range of 120–360 µmol/L. In neonates, Phe intake is established through a gradual titration of breast milk or standard infant formula, supplemented with Phe-free formula until the target range is reached. Once individual tolerance is established, dietary adjustments are required only when values are repeatedly outside the target range [10].

Evidence on gut microbiota in infants and toddlers with PKU is very limited and inconclusive: a pilot study reported no significant differences compared to healthy controls [158], while a preliminary study in older children observed compositional changes highlighting the lack of robust data in early life [159]. Other studies in children and adolescents with PKU have shown that a restrictive diet can alter the gut microbiota, reducing microbial diversity and the abundance of SCFAs–producing bacteria [160,161]. Furthermore, analysis of infant PSs for PKU indicates that few contain microbiota-modulating components [143]. These findings underscore the need for further research to clarify the effects of PKU and dietary management on microbiota development, particularly in early life.

Frequent monitoring of blood Phe, with a recommendation of at least one dried blood spot (DBS) per week, allows effective dietary titration, particularly during periods of rapid infant growth, when the amount of natural protein tolerated changes quickly [162]. There is evidence that patients with PKU can often tolerate more Phe than prescribed [163]. For this reason, when blood Phe values remain consistently at the lower end of the target range but not below 120 µmol/L (e.g., 120–240 µmol/L), it is advisable to test tolerance by increasing dietary Phe in increments of 25–50 mg. This approach helps optimize dietary management, promoting—whenever possible—an increase in natural protein sources, which may also provide functional benefits (e.g., breast milk) [10]. Taken together, these early-life dietary approaches aim not only to maintain metabolic control but also to support long-term growth, neurodevelopment, and overall clinical outcomes in children with PKU.

Although long-term follow-up data are limited, observational evidence suggests that these interventions contribute to metabolic stability and may influence body composition and long-term metabolic risk, highlighting the importance of individualized, closely monitored nutritional strategies.

Table 1 summarises current knowledge and highlights areas for future investigation, categorised into pre- and postnatal periods.

## 4. Discussion

The evidence reviewed highlights that the first 1000 days of life—spanning conception, pregnancy, lactation, and early complementary feeding—represent a critical window for shaping long-term health outcomes in individuals with PKU. Nutritional management during this period is crucial not only in optimizing metabolic control and neurodevelopmental trajectories, but also in establishing lifelong dietary habits that may influence the risk of NCDs [3]. Suboptimal Phe control during early life may induce metabolic adaptations, including altered glucose and lipid metabolism, that could predispose individuals with PKU to NCDs in adulthood [165]. Emerging evidence also suggests that gut microbiota composition, which can be influenced by early dietary interventions, may mediate some of these long-term metabolic effects [61]. Further research is needed to clarify the role of gut microbiota and epigenetic mechanisms in shaping metabolic outcomes in PKU.

Given the global increase in population aging, comorbidities, and chronic conditions such as obesity, cardiovascular disease, type 2 diabetes, and dyslipidaemia, early nutritional intervention acquires even greater relevance also in PKU [166,167,168]. Recent longitudinal evidence indicates a progressive increase in overweight prevalence among individuals with PKU, particularly in females and with advancing age [16]. Higher total and natural protein intakes appear to exert a protective effect against overweight, while suboptimal metabolic control and reduced physical activity may exacerbate this risk, underscoring the need for continuous nutritional monitoring and individualized dietary management throughout life [16].

In addition, current evidence emphasizing the importance of early-life nutrition in PKU highlights that SLPFs contribute substantially to overall macronutrient intake, supplying approximately one-third of total energy in children with early-treated PKU [169]. While SLPFs effectively support energy requirements within Phe-restricted diets, enhancing their nutritional composition—particularly fibre quality and micronutrient density—remains essential to promote optimal long-term metabolic health [164]. Suboptimal dietary adherence and the inadequate composition of Foods for Special Medical Purposes (FSMPs) may lead to unfavourable body composition, increased fat mass, and metabolic disturbances later in life. Recent evidence underscores the need to continuously improve the nutritional quality of FSMPs, including both PSs and SLPFs, through formulations that better mimic natural protein sources, enhance satiety, and support optimal growth without promoting excessive energy intake [143].

Recent advances in pharmacological therapies for PKU, now also available during pregnancy, have expanded the therapeutic options for maintaining metabolic control while allowing for a higher intake of natural protein [11]. Evidence on the use of pharmacological therapies, such as BH4 and Pegvaliase, during pregnancy is still limited and largely based on small case series. These treatments facilitate greater dietary flexibility and may improve adherence; however, dietary liberalization does not inherently ensure optimal nutritional quality. Further research is needed to establish their safety, efficacy, and long-term clinical outcomes for both mother and offspring. Careful monitoring of nutrient adequacy and overall diet composition remains essential to safeguard maternal and foetal health and to sustain optimal metabolic outcomes during this critical developmental window, with potential long-term benefits in reducing the risk of NCDs.

Psychological support during the first 1000 days in PKU is a crucial component of comprehensive care, both for women during pregnancy and for families receiving a new diagnosis [170,171]. Early psychological counselling helps parents cope with the emotional burden of managing a chronic metabolic condition, facilitates adaptation to the demanding dietary regimen, and supports the establishment of healthy feeding practices from infancy. For pregnant women with PKU, ongoing psychosocial support contributes to better treatment adherence, stress reduction, and improved metabolic control, ultimately promoting optimal neurodevelopmental outcomes for the child [172].

Finally, home monitoring of plasma Phe is a rapidly growing area in PKU management. It is particularly valuable during pregnancy, when metabolic demands fluctuate, and in the first two years of life, when rapid growth can alter Phe tolerance. Recent advances, including point-of-care devices, allow more frequent and flexible assessment of metabolic control, enabling timely dietary adjustments, reducing hospital visits, and supporting optimal neurodevelopmental and maternal-foetal outcomes [173,174].

Based on the current evidence, several practical clinical priorities emerge for the first 1000 days in PKU. Early and individualized dietary management, including careful titration of natural protein, use of Phe-free PSs, and timing of CF, is critical to optimise metabolic control and support neurodevelopment. Frequent monitoring of blood Phe, particularly during pregnancy and periods of rapid infant growth, enables timely dietary or pharmacological adjustments [175,176]. Attention to nutrient adequacy and quality of PSs and SLPFs is essential to support optimal growth, body composition, and long-term metabolic health. Psychological and social support for families and pregnant women is fundamental to improve adherence, reduce stress, and facilitate healthy feeding practices [177,178,179]. Finally, emerging pharmacological therapies may allow greater dietary flexibility but must be combined with careful nutritional monitoring to ensure maternal and foetal safety [180,181]. These priorities emphasize the need for a multidisciplinary approach integrating metabolic, nutritional, and psychosocial care, translating current evidence into actionable clinical recommendations.

This review has limitations that should be acknowledged. First, as a narrative review, no a priori protocol was registered (e.g., in PROSPERO), and we did not formally assess study quality or risk of bias. Second, the literature search was limited to two databases and only included English-language publications, which may have introduced selection and language bias. Third, evidence from the general population was drawn from authoritative sources but was not identified through a systematic search, which may have affected the completeness of comparisons and should be considered when interpreting our findings. Finally Additionally, the results are presented narratively, reflecting the heterogeneity of the included evidence. Finally, the small sample sizes, heterogeneity of dietary interventions, limited follow-up duration, and predominance of observational studies further constrain the generalizability and strength of the conclusions. Despite these limitations, the structured search approach and transparent reporting support the reliability and clarity of our findings.

**Figure 2:**
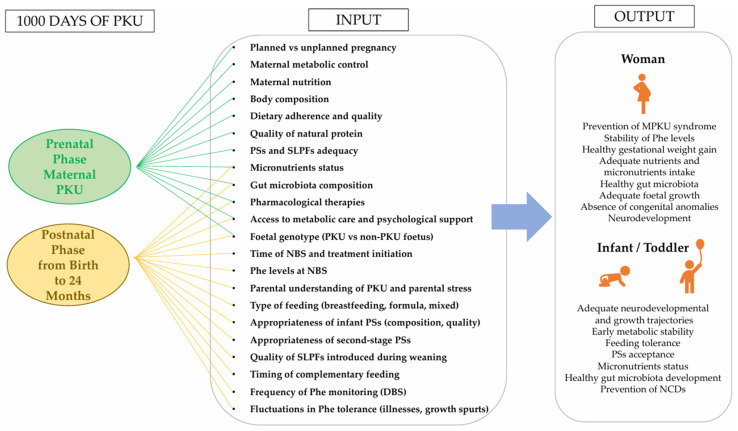
Interplay of key maternal, nutritional, psychosocial, and clinical factors that influence metabolic control and developmental outcomes in PKU throughout the first 1000 days. Abbreviations. PKU: Phenylketonuria; MPKU: Maternal PKU; Phe: Phenylalanine; PSs: Protein Substitutes; SLPFs: Special Low-Protein Foods; DBS: Dried Blood Spot; NBS: Newborn Screening; NCDs: Non-Communicable Diseases.

Figure 2 illustrates the interconnected biological, nutritional, psychosocial, and healthcare factors influencing metabolic control and developmental outcomes in PKU across the first 1000 days.

## 5. Conclusions

The first 1000 days represent a pivotal window to shape lifelong health outcomes in PKU, from maternal metabolic control to early nutritional and psychosocial care after newborn screening. Emerging pharmacological options and improved dietary formulations offer unprecedented opportunities to optimize both metabolic and neurodevelopmental trajectories. Early, individualized intervention, including careful titration of natural protein, supplementation with adequate Phe-free PSs, home monitoring of plasma Phe, and psychological support for families, should be prioritized to ensure optimal outcomes. A multidisciplinary approach, beginning before conception and extending through infancy, is essential to ensure that early intervention in PKU translates into lasting health and well-being. Continued research is needed to strengthen the evidence base and refine clinical strategies, particularly regarding long-term effects of dietary and pharmacological interventions on growth, neurodevelopment, and metabolic risk.

### Future Perspectives

Despite advances in dietary and pharmacological management of PKU, several important gaps remain, particularly during the first 1000 days of life. Future research should prioritise longitudinal and multicentre studies to clarify the long-term impact of early nutritional interventions on growth, neurodevelopment, metabolic health, and risk of NCDs, using standardised and clinically meaningful outcome measures to improve comparability across studies. In pregnancy, there is a need for PSs tailored to maternal use, with appropriate folic acid and DHA content, as well as strategies for micronutrient supplementation and monitoring. Further evidence is required on the safety, efficacy, and long-term outcomes of pharmacological therapies such as Sapropterin, Pegvaliase, and LNAAs. Data on breast milk composition in relation to maternal metabolic control and the effects of diet liberalisation on nutrient intake and foetal outcomes are also limited. During early infancy, the development of stage-specific PSs and age-appropriate SLPFs with improved nutritional quality is essential. Standardised protocols for Phe washout, protein titration, and Phe tolerance thresholds should be explored, alongside strategies to ensure adequate micronutrient intake. Mechanistic studies on gut microbiota may further elucidate the long-term impact of early dietary interventions, particularly in relation to PSs composition, fibre quality, and the potential role of biotics in early life. Finally, psychosocial support for families remains a key component of care, with interventions to improve adherence, facilitate healthy feeding practices, and reduce caregiver burden requiring further development. Addressing these gaps will inform evidence-based guidelines and support individualised strategies to optimise both short- and long-term outcomes in PKU.

## Figures and Tables

**Table 1:** Summary of current knowledge and priorities for future research in PKU management during the first 1000 days.

Period	Evidence/Key Findings	Future Research/Knowledge Gaps
**Prenatal Phase** **Maternal PKU**	- Optimised maternal metabolic control is essential to prevent MPKU syndrome and adverse foetal outcomes [40,41,42,43,44] - Early pre-conception dietary intervention reduces risk of miscarriages and congenital defects [40,41,42,43] - Safe maternal blood Phe target: 120–360 µmol/L [10,11,46] - Dietary management includes natural protein restriction, Phe-free PSs, SLPFs, and micronutrients [10,11,48,49,50] - Protein requirements increase during pregnancy. Tyrosine ≥ 6 g/day via PSs [10,11] - Micronutrients (folate, DHA, iron, iodine, zinc, selenium, vitamin D, LC-PUFAs) are crucial [51,52,53,54,55,56,57,58,59,60,61] - Pharmacological options (Sapropterin) may allow increased natural protein intake if responsive; safety data for Pegvaliase limited [68,69,70,71,72,73,74,75] - Structured education and support for women with PKU and partners improves adherence [45,57,58,59,60,61,62,63,64,65]	- Lack of commercially available PSs specifically formulated for pregnancy and lactation with particular attention to folic acid and DHA content [50,54] - Optimal strategies for micronutrient supplementation and monitoring [51,52,53,54,55,56,57,58,59,60,61] - Further data on breast milk composition in relation to maternal metabolic control [66,67,72] - Impact of diet liberalisation on nutrient intake and foetal outcomes [64,73] - Long-term outcomes of pharmacological treatments in pregnancy (Sapropterin) [68,69,70,73] - Safety and efficacy of LNAAs and Pegvaliase in pregnancy require more evidence [74,75,76] - Effects of MPKU diet on gut microbiota and offspring metabolism [61] - Strategies to improve adherence and dietary quality during pre-conception and pregnancy [45,57,58,59,60,61,62,63,64,65]
**Postnatal Phase** **from Birth to 24 Months**	- Dietary management aims to maintain blood Phe 120–360 µmol/L via breast milk, standard formula or Phe-free infant PS [10,11,140] - Breastfeeding/Infant formula combined with Phe-free infant PS is safe and supports growth, metabolic control, and neurodevelopment [139,140,141,142] - Frequent Phe monitoring (1 DBS/week) allows dietary titration during rapid growth [11,162] - Complementary feeding introduced 17–26 weeks [10,148] PS quality, composition, and transition (stage 1 → 2) are crucial for intake, acceptance, and growth [143,149,150,151,152,153] - Limited data on micronutrient adequacy (DHA, iron, zinc, selenium, vitamin B12, folate, vitamin D) and gut microbiota development [143,154,155,156,158,159,160,161] - Maternal stress and anxiety may affect adherence and feeding practices [129,130,131,132,157]	- Standardised protocols for Phe washout periods and natural protein titration [140] - Develop stage-specific PSs for infants/toddlers [149,150,151,152,153] - Attention should be paid to the P.Eq. to energy ratio, bioactive compounds, essential fatty acids, and lactose content in infant protein substitutes [143,144,145,146,147] - Development of age-appropriate SLPFs with improved nutritional composition [153,164] - Optimal micronutrient supplementation strategies in early infancy [154,155,156] - Longitudinal studies on gut microbiota impact and interventions [158,159,160,161] - Psychosocial interventions to support caregivers and improve adherence [129,130,131,132] - Exploration of flexible Phe tolerance thresholds to optimise natural protein intake [10,163] - Effects of dietary liberalization with pharmacotherapy [10,133,140]

Abbreviations. PKU: Phenylketonuria; MPKU: Maternal PKU; Phe: Phenylalanine; PSs: Protein Substitutes; SLPFs: Special Low-Protein Foods; DHA: Docosahexaenoic Acid; LC-PUFA: Long Chain Polyunsaturated Fatty Acids; DBS: Dried Blood Spot; LNAAs: Large Neutral Amino Acids; P.Eq.: Protein equivalents.

## Data Availability

No new data were created or analysed in this study. Data sharing is not applicable to this article.

## Supplementary Materials

The following supporting information can be downloaded at: https://www.mdpi.com/article/10.3390/nu18020199/s1, Table S1: PICOS criteria for inclusion of studies.; Table S2: Research strategies employed on PubMed/Medline and Scopus databases.
