# Supplementary material for: The First 1000 Days of PKU: A Narrative Review of Maternal PKU and Early Life Management After Positive Newborn Screening
Source: Nutrients. 2026 Jan 8;18(2):199. doi: 10.3390/nu18020199 (PMC12845488; doi:10.3390/nu18020199)
Supplement: Supplementary file 1 [file nutrients-18-00199-s001.zip › nutrients-4078022-supplementary.pdf]

Supplementary Material

Supplementary Table S1. PICOS criteria for inclusion of studies.

TOPIC	Narrative question	P - Population	I – Interventions	C - Comparison/Control	O - Outcomes
**Topic 1**<br><br>**From maternal PKU to a neonate with positive newborn screening**	In pregnant women with PKU, do targeted nutritional interventions compared with standard care affect the neurological development and metabolic outcomes of the neonate?	Pregnant women with PKU and their fetuses/infants	Nutritional interventions for PKU management (restricted-Phe diet, protein substituted, low-protein foods, supplements) during pregnancy	Pregnancies with PKU not optimally managed or receiving standard care	Identified through:<br><br>- Neurological: cognitive and neurological development of the newborn<br><br>Metabolic: blood phenylalanine levels, risk of congenital malformations, risk for non-communicable disease later in adult life
**Topic 2**<br><br>**The First 1000 days: the Postnatal Phase, from birth to second year of life**	In neonates, infants and young toddlers with PKU during the two first years of life, do targeted nutritional interventions compared with standard care influence neurological and metabolic outcomes?	Neonates, infants and young toddlers up to 2 years old with PKU	Nutritional interventions for PKU management (restricted-Phe diet, protein substituted, low-protein foods, supplements) during childhood	Absence of optimized nutritional interventions or standard care	Identified through:<br><br>- Neurological: cognitive, motor, and behavioral development<br>- Metabolic: phenylalanine level stability, growth, nutritional status and risk for non-communicable disease later in adult life

Supplementary Table S2. Research strategies employed on PubMed/Medline and Scopus databases.

**1.**	**From maternal PKU to a neonate with positive newborn screening – 921 results**
a)	(Phenylketonurias[MeSH] OR phenylketonuria OR PKU) AND (Pregnancy[MeSH] OR pregnant OR "pregnant women") AND (Diet Therapy[MeSH] OR nutrition OR diet OR "nutritional intervention" OR "medical food") – 726 results
b)	(Phenylketonurias[MeSH] OR Phenylketonuria OR PKU) AND (Pregnancy[MeSH] OR pregnant OR "pregnant women") AND (Infant, Newborn[MeSH] OR neonate OR newborn OR "fetal development") AND (Neurological Manifestations[MeSH] OR Cognition[MeSH] OR Neurodevelopmental Disorders[MeSH] OR "cognitive development" OR neurodevelopment OR "brain development" OR Metabolism[MeSH] OR "metabolic outcomes" OR "metabolic control" OR "phenylalanine levels") – 195 results
**2.**	**The First 1000 days: the Postnatal Phase, from birth to second year of life – 461 results**
a)	(Phenylketonurias[MeSH] OR phenylketonuria OR PKU) AND (Infant[MeSH] OR "infant" OR "infants" OR "toddler" OR "toddlers" OR "young child" OR neonate OR newborn) AND (Diet Therapy[MeSH] OR diet OR nutrition OR "nutritional intervention" OR "medical food") AND (Neurological Manifestations[MeSH] OR Cognition[MeSH] OR Neurodevelopmental Disorders[MeSH] OR "cognitive development" OR neurodevelopment OR "brain development" OR Metabolism[MeSH] OR "metabolic outcomes" OR "metabolic control" OR "phenylalanine levels") – 461 results
